# Chronic obstructive pulmonary disease and comorbid psychiatric disorders: preliminary results of an 8-year retrospective study using real data

**DOI:** 10.1192/j.eurpsy.2023.1091

**Published:** 2023-07-19

**Authors:** G. Santos, A. R. Ferreira, M. Gonçalves-Pinho, A. Freitas, L. Fernandes

**Affiliations:** 1 Faculty of Medicine, University of Porto (FMUP), Porto, Portugal; 2 CINTESIS@RISE, Department of Clinical Neurosciences and Mental Health, Faculty of Medicine, University of Porto (FMUP), Porto; 3 Department of Psychiatry and Mental Health, Centro Hospitalar do Tâmega e Sousa, Penafiel; 4 CINTESIS@RISE, Department of Community Medicine, Information and Health Decision Sciences (MEDCIDS), Faculty of Medicine, University of Porto (FMUP); 5 Psychiatry Service, Centro Hospitalar Universitário de São João, Porto, Portugal

## Abstract

**Introduction:**

Chronic obstructive pulmonary disease (COPD) is the third leading cause of mortality worldwide. In Portugal, it is estimated to afflict 14.2% of the population over the age of 45 and is one of the most common causes of morbidity, with a significant social impact and excessive expenses. Moreover, COPD is associated with high levels of psychological distress and diverse psychiatric disorders that heighten the disease burden as they are associated with increased risk of exacerbations and frequent hospitalizations. Despite this overview, psychiatric conditions remain understudied compared to comorbid general medical conditions, and few studies have assessed their effect on COPD hospitalization outcomes.

**Objectives:**

This study aimed to describe the occurrence of a vast array of psychiatric comorbid diagnoses in COPD hospitalizations and to understand their impact on hospitalization outcomes.

**Methods:**

A retrospective observational study was conducted. All inpatient episodes from 2008 to 2015 of patients with at least 40 years and a primary diagnosis of COPD (ICD-9-CM codes 491.x, 492.x and 496) were selected from a national administrative database that included all hospitalizations in mainland public hospitals. From these sampled episodes, secondary psychiatric diagnoses were identified (ICD-9-CM codes 290.x-319.x). Age at hospitalization, sex, psychiatric comorbidities, length of stay (LoS) in days, admission type and date, destination after discharge, in-hospital mortality and hospital charges were analyzed.

**Results:**

From a total of 66,661 COPD hospitalizations, 17,652 (26.5%) corresponded to episodes with a secondary psychiatric diagnosis. Patients with a comorbid psychiatric diagnosis were on average younger at admission (70.3 vs. 75.9 years, p<0.001), had a longer median LoS (9.89 vs. 9.33 days, p<0.001) and higher urgent admission rates (96.2% vs. 95.7%, p=0.009). There was also a significant association between discharge destination and psychiatric diagnoses (p<0.001).

**Conclusions:**

These findings suggest that mental disorders have an adverse and quantifiable impact on COPD hospitalization outcomes. With this in mind, to provide optimal treatment for patients with both conditions, psychiatric disorders should become a matter of routine evaluation and follow-up.

**Disclosure of Interest:**

None Declared

